# Risk Factors of Neurosensory Disturbance following Orthognathic Surgery

**DOI:** 10.1371/journal.pone.0091055

**Published:** 2014-03-05

**Authors:** Albraa Badr Alolayan, Yiu Yan Leung

**Affiliations:** Oral and Maxillofacial Surgery, Faculty of Dentistry, The University of Hong Kong, Hong Kong, Hong Kong; Georgia Regents University, College of Dental Medicine, United States of America

## Abstract

**Objectives:**

To report the incidence of objective and subjective neurosensory disturbance (NSD) after orthognathic surgery in a major orthognathic centre in Hong Kong, and to investigate the risk factors that contributed to the incidence of NSD after orthognathic surgery.

**Materials and Methods:**

A retrospective cross-sectional study on NSD after orthognathic surgery in a local major orthognathic centre. Patients who had bimaxillary orthognathic surgery reviewed at post-operative 6 months, 12 months or 24 months were recruited to undergo neurosensory tests with subjective and 3 objective assessments. Possible risk factors of NSD including subjects’ age and gender, surgical procedures and surgeons’ experience were analyzed.

**Results:**

238 patients with 476 sides were recruited. The incidences of subjective NSD after maxillary procedures were 16.2%, 13% and 9.8% at post-operative 6 months, 12 months and 24 months, respectively; the incidences of subjective NSD after mandibular procedures were 35.4%, 36.6% and 34.6% at post-operative 6 months, 12 months and 24 months, respectively. Increased age was found to be a significant risk factor of NSD after orthognathic surgery at short term (at 6 months and 12 months) but not at 24 months. SSO has a significantly higher risk of NSD when compared to VSSO. SSO in combination with anterior mandibular surgery has a higher risk of NSD when compared to VSSO in combination with anterior mandibular surgery or anterior mandibular surgery alone. Gender of patients and surgeons’ experience were not found to be risk factors of NSD after orthognathic surgery.

**Conclusion:**

The incidence of NSD after maxillary and mandibular orthognathic procedures at post-operative 6 months, 12 months and 24 months was reported. Increased age was identified as a risk factor of short term post-operative NSD but not in long term (24 months or more). Specific mandibular procedures were related to higher incidence of NSD after orthognathic surgery.

## Introduction

Neurosensory disturbance (NSD) is one of the most common post-operative complications following orthognathic surgery. While many patients with dentofacial deformities benefitted from the drastic functional and aesthetic improvement after orthognathic surgery, they could be quite bothered by the post-operative NSD of the lips and facial region. Patients with post-operative NSD may present with hypoaesthesia, anaesthesia or dysaesthesia of the facial region supplied by the affected trigeminal nerve branches [1]. Although a systematic review showed 12.8% of the patients had persistent NSD by objective measurement after an orthognathic surgical procedure [2], the reported incidences of subjective NSD after orthognathic surgery varied in the literature and could be as high as 87% [1], [3], [4], [5], [6], [7], [8], [9], [10], [11]. Reports on the incidences of NSD after maxillary orthognathic procedures were few, and the knowledge on NSD after complex multisegmental orthognathic procedures was also insufficient in the literature. Risk factors like patients’ age, surgical procedures and surgeons’ experience have been suggested that might contribute to NSD after orthognathic surgery [12], [13], [14]. However, with the limitation of the scale of the studies and the lack of standardized evaluations, there is yet a solid answer to the clinical question “what are the risk factors of NSD after orthognathic surgery?”.

The purposes of this study were to report the incidence of objective and subjective NSD after orthognathic surgery in a major orthognathic centre in Hong Kong, and to investigate the risk factors that contributed to the incidence of NSD after orthognathic surgery.

## Materials and Methods

### Study Design and Sampling

This was a retrospective cross-sectional study to investigate the incidence of NSD and its risk factors after orthognathic surgery. The inclusion criteria were patients with dentofacial deformities who had bimaxillary orthognathic surgery between September 2009 and January 2013 in Queen Mary Hospital under the care of the Discipline of Oral and Maxillofacial Surgery, Faculty of Dentistry, The University of Hong Kong. The exclusion criteria were 1.Patients with pre-existing trigeminal NSD; 2. Intra-operatively the inferior alveolar nerve, the mental nerve, or the infra-orbital nerve was transected by accident; 3. Patients presenting with infection or plate exposure at the time of review. The study protocol was approved by the Institutional Review Board of the University of Hong Kong/Hospital Authority Hong Kong West Cluster (Protocol no. UW 12–200). All patients signed study consent to participate in the study.

### Data Collection

Demographic (age and gender) data of the patients were recorded. The clinical diagnoses of the patients’ dentofacial deformity (maxilla and mandible) were recorded. The maxillary diagnoses included 1. Maxillary hypoplasia; 2. Maxillary hypoplasia with dentoalveolar hyperplasia; or 3. Dentoalveolar hyperplasia. The mandibular diagnoses included 1. Mandibular hypoplasia; 2. Mandibular hyperplasia; 3. Mandibular hypoplasia with dentoalveolar hyperplasia; 4. Mandibular hyperplasia with dentoalveolar hyperplasia; or 5. Dentoalveolar hyperplasia. Patients were classified with one maxillary diagnosis and one mandibular diagnosis.

The surgical procedures the patients received were recorded. Maxillary procedures included LeFort 1 osteotomy in 1 piece, LeFort 1 osteotomy in 2 pieces, or LeFort 1 osteotomy in 4 pieces. Mandibular procedures included sagittal split osteotomy (SSO) for mandibular advancement, vertical subsigmoid osteotomy (VSSO) for mandibular setback or decanting, anterior mandibular surgery (including anterior subapical osteotomy and/or genioplasty), or a combination of the above.

The surgeons’ experiences of the specific surgical procedure were classified as resident (within 3 years of training, with experience of 0–40 surgical procedures), senior resident (3–6 years of training, with experience of 40–100 surgical procedures) or specialist (6 or more years of surgical experience who performed over 100 surgical procedures) and were recorded.

A standardized neurosensory test was performed on all study participants at their follow-up appointments at post-operative 6 months, 12 months and 24 months. The neurosensory test was performed on *each quadrant* of the face which represented the sensory distribution of infra-orbital nerve (at the infra-orbital region) and inferior alveolar nerve/mental nerve (at the mental region).

#### Neurosensory test

The neurosensory test included a subjective assessment and three objective assessments. For the subjective assessment, the patient was asked to rate the NSD of the area on the face by a visualized analog scale (VAS) from 0 (normal sensation) to 10 (most severe sensory deficit). Three objective assessments were performed on each participants on each operated side described as follow:

1. Static light touch threshold
  Von Frey fibres of ascending diameter were applied to the facial quadrant and the lightest fibre (in terms of gram of force to bend the fibre) a patient could feel was recorded. When the tip of a fibre of given length and diameter was pressed against the skin at right angles, a definitive force was applied when the fibre bent. The set consists of 20 fibres of different diameters. The sizes of the filaments were in the approximate logarithm scale of the actual forces listed in **Table 1**:
2. Two-point discrimination
  A set of paired blunt metallic probes of 0.8 mm diameter with separations ranging from 2–20 mm at 2 mm interval were applied with a constant force to the facial quadrant at an ascending order. The smallest separation of the probes that a patient could discriminate a two-point sensation was recorded.
3. Pain threshold
  A blunted 19G needle connected to a spring gauge was applied to the facial quadrant until the patient starts to feel “pain” as the force gradually increased and the force in terms of gram was recorded. Three readings were taken and the mean was recorded.

**Table 1:** Actual force in gram applied in relation to the various sizes of the Von Frey fibres.

**Size**	1.65	2.36	2.44	2.83	3.22	3.61	3.84	4.08	4.17	4.31
**Force (in g)**	0.008	0.02	0.04	0.07	0.16	0.4	0.6	1	1.4	2
**Size**	4.56	4.74	4.93	5.07	5.18	5.46	5.88	6.1	6.45	6.65
**Force (in g)**	4	6	8	10	15	26	60	100	180	300

### Outcome Measures

The outcomes of the study were to report the incidence and severity of subjective and objective neurosensory disturbances after orthognathic surgery at post-operative 6 months, 12 months and 24 months, and to investigate possible risk factors (patients’ age and gender, surgical procedures and surgeons’ experience) that might contribute to the presence of the subjective NSD after orthognathic surgery.

### Statistical Analysis

The data were analyzed with the Statistical Package for Social Sciences (SPSS version 20.0 SPSS Inc, Chicago, IL, USA). The proportion of sample presented with NSD after maxillary and mandibular orthognathic surgical procedures were compared with different gender of patients, surgical procedures performed and surgeons’ experience by chi-square tests at different post-operative time. The mean age of the subjects and the presence of objective neurosensory test readings versus the presence of subjective NSD were compared with independent t-tests at different post-operative time. A 5% level of significance was applied.

## Results

**Figure 1:**
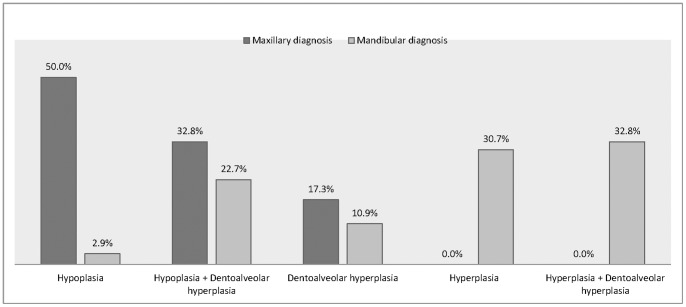
Dentofacial deformity diagnoses of study participants (n = 238).

There were 238 patients with 476 sides each of maxillary and mandibular procedures recruited in the study. There were 105 patients (210 sides), 82 patients (164 sides) and 51 patients (102 sides) attended the follow-up period at post-operative 6 months, 12 months and 24 months, respectively. The mean age of the subjects was 25.1 years (S.D. 5.6 years). 37.8% (90/238) of the patients were male. The maxillary and mandibular diagnoses of the subjects were presented in **Figure 1**.

The maxillary and mandibular surgical procedures that the subjects received and the surgeons’ experience were presented in **Table 2**. More than half (50.4%) of the subjects had LeFort 1 in four pieces as the maxillary procedures. For mandibular procedures, 89% of the subjects had ramus surgeries (SSO and/or VSSO) and 87% of the subjects had anterior mandibular surgery (anterior subapical osteotomy and/or genioplasty). In terms of the surgeons’ experience, 76% of the procedures were performed by residents or senior residents, and about 24% were performed by specialists.

**Table 2:** Surgery related data of the subjects reviewed at various post-operative times.

			6 Months (n = 210)	12 Months (n = 164)	24 Months (n = 102)	Overall
**Surgery**	*Maxillary procedure*	Le Fort 1	26.5%	19.5%	29.4%	58 (24.4%)
		Le Fort 1 in 2 pieces	28.8%	27%	15.6%	60 (25.2%)
		Le Fort 1 in 4 pieces	45%	53.5%	55%	120 (50.4%)
	*Mandible procedure* *	SSO	2%	1.3%	0	5 (2.1%)
		VSSO	13.4%	11%	10%	26 (10.9%)
		Anterior mandibular surgery	14.4%	9.7%	6%	26 (10.9%)
		SSO+ Anterior mandibular surgery	19.2%	30.5%	17.5%	56 (23.5%)
		VSSO+ Anterior mandibular surgery	51%	47.5%	66.5%	125 (52.5%)
**Surgeons’ experience**		Resident	42.4%	39.6%	35.3%	110 (46.2%)
		Senior Resident	16.6%	22%	29.4%	71 (29.8%)
		Specialist	41%	38.4	35.3%	57 (24.0%)

*SSO: Sagittal split osteotomy; VSSO: vertical subsigmoid osteotomy; Anterior mandibular surgery: Anterior subapical osteotomy and/or genioplasty).

The incidences and severity of NSD at different post-operative times were presented in **Table 3**. The incidences of neurosensory disturbance of maxilla (by sides) at post-operative 6 months, 12 months and 24 months were 16.2%, 13% and 9.8%, respectively. 35% of the maxillary sides presented with subjective moderate to severe NSD at post-operative 6 months, which was reduced to 19% of maxillary sides with moderate neurosensory disturbance at post-operative 12 months and a similar proportion at post-operative 24 months. The incidences of NSD of mandible (by sides) at post-operative 6 months, 12 months and 24 months were 35.4%, 36.6% and 34.6%, respectively. There were similar proportions of subjects presented with NSD in the mandibular sides in terms of subjective severity. The findings of the objective neurosensory assessments at various post-operative times in subjects presenting with NSD were presented in **Table 4**. In general, those who complained of subjective NSD had a reduced sensitivity in static light touch threshold, two-point discrimination and pain threshold when compared to those who did not have perceived NSD, with several of these comparisons at various time-points were found to be statistically different.

**Table 3:** Incidences of subjective neurosensory disturbance.

		6 Months (n = 210)	12 Months (n = 164)	24 Months (n = 102)
**Maxilla**	***Incidence of NSD***	16.2%	13%	9.8%
	***Severity***			
	Mild	65%	81%	80%
	Moderate	32%	19%	20%
	Severe	3%	0	0
	Mean VAS 0–10 (S.D.)	3.3(2.0)	2.5(1.2)	2.6(1.8)
**Mandible**	***Incidence of NSD***	35.4%	36.6%	34.6%
	***Severity***			
	Mild	51.3%	43%	50%
	Moderate	38.4%	57%	44.4%
	Severe	10.3%	0	5.6%
	Mean VAS 0–10 (S.D.)	3.8(2.3)	4.1(1.7)	3.6(2.1)

**Table 4:** Comparisons of the objective assessment findings in subjects with or without subjective neurosensory disturbance.

		6 Months			12 Months			24 Months		
		*Presence of NSD*			*Presence of NSD*			*Presence of NSD*		
		Yes	No	*p value*	Yes	No	*p value*	Yes	No	*p value*
**Maxilla**	*Static light touch threshold*	1.76 (S.D. 0.26)	1.70 (S.D. 0.19)	0.133	1.77 (S.D. 0.28)	1.67 (S.D. 0.14)	0.064	1.68 (S.D. 0.15)	1.65 (S.D. 0.00)	0.449
	*Two-point discrimination(mm)*	9.0 (S.D. 1.9)	9.2 (S.D. 2.7)	0.626	9.2 (S.D. 2.5)	9.0 (S.D. 4.0)	0.835	10.6 (S.D. 2.7)	9.1 (S.D. 4.1)	0.005
	*Pain threshold(g)*	44.4 (S.D. 48.4)	33.4 (S.D. 15.8)	0.017	35.8 (S.D. 11.0)	30.8 (S.D. 12.5)	0.164	42.1 (S.D. 24.8)	30.8 (S.D. 12.6)	0.037
**Mandible**	*Static light touch threshold*	1.81 (S.D. 0.36)	1.73 (S.D. 0.25)	0.072	1.78 (S.D. 0.37)	1.67 (S.D. 0.12)	0.005	1.75 (S.D. 0.28)	1.68 (S.D. 0.15)	0.137
	*Two-point discrimination (mm)*	9.2 (S.D. 3.8)	8.3 (S.D. 3.9)	0.068	10.0 (S.D. 4.2)	8.6 (S.D. 4.7)	0.065	11.2 (S.D. 3.8)	9.2 (S.D.3.6)	0.01
	*Pain threshold (g)*	35.4 (S.D. 15.1)	34.6 (S.D. 17.9)	0.769	37.3 (S.D. 16.9)	32.3 (S.D. 13.9)	0.043	37.5 (S.D. 19.7)	34.6 (S.D. 15.0)	0.398

The possible risk factors of NSD after orthognathic surgery including patient’s age and gender, surgical procedures and surgeons’ experience were analyzed. It was found that older age was a significant risk factor of NSD after orthognathic surgery for maxillary procedures at post-operative 6 months (mean age 26.1 years (S.D. 5.3 years) of subjects with NSD versus mean age 23.8 years (S.D. 4.5 years) in those without NSD, p = 0.011) and 12 months (mean age 27.7 years (S.D. 7.2 years) of subjects with NSD versus mean age 24.6 years (S.D. 4.7 years) in those without NSD, p = 0.011). There was no statistical difference in the mean age with or without NSD at post-operative 24 months. Older age was also a significant risk factor of NSD for mandibular procedures at post-operative 6 months (mean age 25.0 years (S.D. 3.1 years) with NSD versus mean age 23.7 years (S.D. 4.5 years) without NSD, p = 0.042). No statistical differences were found in age at post-operative 12 months and 24 months in subjects who received mandibular procedures. There were also no statistical differences in the incidences of NSD between genders and the surgeons’ experience at all post-operative times (**Table 5**).

**Table 5:** Mean age, gender and surgeon’s experience and the incidence of neurosensory disturbance.

	6 Months (n = 210)		12 Months (n = 164)		24 Months (n = 102)	
	Incidence	p value	Incidence	p value	Incidence	p value
**Gender**						
*Maxilla*		0.320		0.567		0.239
Male	13%		11%		14%	
Female	18.3%		14%		7%	
*Mandible*		0.262		0.479		0.735
Male	31.1%		33.3%		32.3%	
Female	38.4%		38.6%		35.7%	
**Surgeon’s Experience**		0.191		0.164		0.541
*Maxilla*						
Resident	21.5%		16.9%		11%	
Senior Resident	13.5%		16.2%		13%	
Specialist	11.7%		6.4%		5.5%	
*Mandible*		0.247		0.882		0.530
Resident	37.6%		38.2%		36.1%	
Senior Resident	44.4%		37.8%		26.6%	
Specialist	18%		34.3%		39.4%	
**Mean Age (S.D.)**						
*Maxilla*		0.011		0.011		0.573
With NSD	26.1 years (5.3 years)		27.7 years (7.2 years)		27.5 years (1.7 years)	
Without NSD	23.8 years (4.5 years)		24.6 years (4.7 years)		26.3 years (6.1 years)	
*Mandible*		0.042		0.112		0.496
With NSD	25.0 years (3.1 years)		26.2 years (6.5 years)		27.2 years (6.3 years)	
Without NSD	23.7 years (4.5 years)		24.8 years (4.9 years)		26.2 years (6.6 years)	

There were more subjects (30.3%) presented with subjective NSD at post-operative 6 months after receiving LeFort 1 in one piece as the maxillary procedure when compared with those who had LeFort 1 in two pieces (10%) or in four pieces (11.7%) (p = 0.003). There were no statistical differences in the incidence of NSD between subjects receiving different maxillary procedures at post-operative 12 months and 24 months (**Table 6**).

**Table 6:** Maxillary orthognathic procedures and the incidence and severity of neurosensory disturbance.

	6 Months (n = 210)				12 Months (n = 164)				24 Months (n = 102)			
	LeFort 1	Le Fort 1in 2 pieces	Le Fort 1in 4 pieces	*p value*	LeFort 1	Le Fort 1in 2 pieces	Le Fort 1in 4 pieces	*p value*	LeFort 1	Le Fort 1in 2 pieces	Le Fort 1in 4 pieces	*p value*
**Incidence of** **NSD**	30.3%	10%	11.7%	0.003	3.1%	16%	14%	0.185	3.3%	6.2%	14.2%	0.232
**Severity**												
Mild	58.8%	83.3%	63.6%		0	85.7%	84.6%		100%	100%	75%	
Moderate	35.2%	16.7%	36.4%		100%	14.3%	15.4%		0	0	25%	
Severe	6%	0	0		0	0	0		0	0	0	

The NSD after mandibular procedures was compared (**Table 7**). For the ramus surgeries alone, there were significantly more subjects presented with subjective NSD in SSO when compared to VSSO at post-operative 12 months (p>0.001) and 24 months (p>0.001). The combinations of ramus surgeries with anterior mandibular surgery were compared with anterior mandibular surgery alone. It was noted that there were significantly more subjects who had SSO combined with anterior mandibular surgery presented with NSD than those who received only anterior mandibular surgery at post-operative 24 months (p = 0.004). The combination of VSSO and anterior mandibular surgery did not increase the neurosensory risk significantly when compared to those who had anterior mandibular surgery alone at all post-operative times. Subjects who had a combination of SSO and anterior mandibular surgery had NSD significantly more than those who had a combination of VSSO and anterior mandibular surgery at all post-operative times (p<0.05). When comparing ramus surgeries alone with anterior mandibular surgeries alone, it was found that SSO had significantly higher incidences of NSD at post-operative 12 months (p = 0.008) and 24 months (p = 0.005). In contrast, there were no statistical differences of NSD incidences at all follow-up times between VSSO and anterior mandibular surgeries.

**Table 7:** Mandibular orthognathic procedures and the incidence and severity of neurosensory disturbance.

	6 Months (n = 210)					12 Months (n = 164)					24 Months (n = 102)				
	SSO (n = 5)	VSSO (n = 27)	Ant. Mand (n = 32)	*SSO+ Ant.* *Mand (n = 48)*	*VSSO+Ant.* *Mand (n = 108)*	SSO (n = 5)	VSSO (n = 19)	Ant. Mand (n = 18)	*SSO+ Ant.* *Mand (n = 52)*	*VSSO+Ant.* *Mand (n = 78)*	SSO (n = 2)	VSSO (n = 10)	*Ant.Mand (n = 6)*	*SSO+Ant.* *Mand (n = 19)*	*VSSO+ Ant.* *Mand (n = 67)*
**Incidence of NSD**	60%	29.6%	31.2%	48%	31.4%	100%	10.5%	33.3%	59.6%	24.3%	100%	0	0	68.4%	31.3%
**Severity**															
Mild	66.6%	62.5%	70%	26%	58.8%	40%	50%	100%	29%	47.3%	100%	0	0	15.4%	66.7%
Moderate	0	12.5%	30%	56.5%	38.2%	60%	50%	0	71%	52.7%	0	0	0	69.2%	33.3%
Severe	33.4%	25%	0	7.5%	3%	0	0	0	0	0	0	0	0	15.4%	0
***Comparisons***															
	***6 Months***					***12 Months***					***24 Months***				
**Surgery**	***p value***					***p value***					***p value***				
**SSO** *versus* **VSSO**	0.189					0.000					0.001				
**SSO+Ant Mand** *versus* **VSSO+Ant. Mand**	0.049					0.000					0.004				
**SSO+Ant. Mand** *versus* **Ant. Mand**	0.138					0.054					0.003				
**VSSO+Ant Mand** *versus* **Ant. Mand**	0.980					0.434					0.104				
**SSO** *versus* **Ant. Mand**	0.210					0.008					0.005				
**VSSO** *versus* **Ant. Mand**	0.821					0.092					–				

*SSO: Sagittal split osteotomy; VSSO: vertical subsigmoid osteotomy; Ant. Mand: Anterior mandibular surgery (anterior subapical osteotomy and/or genioplasty).

## Discussion

This study reported the incidence of NSD after maxillary and mandibular orthognathic surgery and the severity at post-operative 6 months, 12 months and 24 months. In the literature, the majority of the studies on NSD after orthognathic surgery were on mandibular procedures and relatively few on maxillary procedures. NSD was reported to be less frequent after maxillary procedures than the mandibular procedures [5]. It might be due to the cause of NSD in maxillary procedures is usually nerve retraction while nerve involvement within osteotomy segments occasionally occurs in mandibular surgeries, especially in sagittal split osteotomy,which might cause more trauma to the nerve bundle [6]. It was reported the incidence of permanent NSD after maxillary procedures ranged from 0 to 6% [5], [7], [8]. In our study, we noted 13% and 9.8% of NSD at post-operative 12 months and 24 months, respectively. The majority (around 80%) of the NSD were only rated mild by the subjects, with none reported to have a severe deficit. These findings concurred with the literature of the low incidence of NSD in long term after maxillary procedures, and even if it happened, it was unlikely to be bothersome to the patients. On the other hand, mandibular procedures were known to cause more NSD than the maxillary counterparts. Persistent NSD was reported as high as 87% after SSO [9], although a lot of the studies reported the likely figure of permanent NSD after SSO would be around 20–40% [11]–[13]. Genioplasty is also a mandibular procedure that contributed to the higher incidence of NSD due to the close proximity of the osteotomy and the mental loop of the nerve [12], [14]. Our study showed a similar incidence of 34.6% at post-operative two years when compared to the literature. Moreover, the severity of the NSD was found to be more significant after mandibular procedures, with over 40% of subjects with NSD reported the magnitude to be moderate and 5.6% to be severe at 24 months. This potentially might affect the quality of life of the patients as in those with NSD caused by other oral surgical procedures [15], [16].

Objective neurosensory tests were used in many studies on NSD after orthognathic surgeries^ 1,^ [12], [13]. Many tests for neurosensory monitoring after orthognathic surgery have been suggested but there appeared to have a lack of consent of which test(s) was most suitable to represent the actual NSD the patient suffered [13]. The three objective tests we used in general could show reduced sensation in subjects who complained of NSD. However, we inclined to agree with Essick et al. who suggested clinical judgment of NSD should not be based on threshold testing without consideration of patients’ subjective report of altered sensation [1].

Our study identified increased age was a risk factor of NSD after orthognathic surgery in short term (6 months and 12 months after maxillary procedures, 6 months after mandibular procedures). Previous studies in the literature described age as a risk factor of persistent NSD after orthognathic surgery [9], [11], [17], [18]. Nesari et al. suggested the difference in the bony architecture in older patients leaded to a different split pattern in SSO which might account for the higher NSD [11]. August et al. hypothesized freeing the inferior alveolar nerve from the proximal bony segment after SSO might be more difficult in older patients, which possibly might traumatize the neurovascular bundle to a bigger extent [9]. Our study noted older subjects were correlated with NSD after maxillary procedures at post-operative 6 months and 12 months, but no statistical difference in age at 24 months. We therefore suggest that neurosensory recovery may be faster in younger patients. However, the long term outcome (24 months or more) NSD recovery may not be age-related.

Gender has not been shown to affect the incidence of NSD after orthognathic surgery in previous studies [14], [18], [19]. Our study was in line with the previous studies that gender was not found to be a risk factor of NSD in maxillary or mandibular orthognathic procedures.

The facial profiles and deformities of our population are different from the Caucasian and the northern Chinese population [20], [21]. There are two characteristics/deformities in our local patient group which seems to be more prevalent than many major orthognathic centres: 1. Mandibular prognathism with or without asymmetry; 2. Dentoalveolar hyperplasia leading to protrusive upper and/or lower lips. There were over 63.5% of our subjects presented with mandibular hyperplasia, and 66.4% presented with a component of dentoalveolar hyperplasia. In our centre we perform VSSO when mandibular setback is required to correct mandibular hyperplasia with or without asymmetry, which may improve stability when dealing with moderate to severe canting or changing the occlusal plane. Our study have also shown the risk of having persistent NSD (12 months or more) was significantly lower after VSSO when compared to SSO. It is logical to deduce the difference in the NSD incidences between the two ramus surgeries was from the likely involvement of inferior alveolar nerve during a split of SSO. To correct the dentoalveolar hyperplasia, LeFort 1 in four pieces were usually performed to upright the maxillary anterior segments, which could also allow sufficient advancement in maxillary hypoplasia cases. Anterior subapical osteotomy was the workhorse to correct mandibular dentoalveolar hyperplasia and was performed in 86.9% of the subjects in our study who received mandibular surgery. However, multi-segmental maxillary or mandibular orthognathic procedures have the drawbacks of increasing the complexity and surgical time of the surgery when compared to the conventional one piece maxillary and/or mandibular procedures, which are more popular in many major orthognathic centres. It was also shown in our study that complex mandibular procedures involving anterior subapical osteotomy and/or genioplasty with SSO significantly increase of the risk of post-operative NSD. Kim et al. reported a similar finding of greater extent of NSD in patients who received genioplasty [14]. The close proximity of the mental nerves and the surgical sites in anterior mandibular surgeries may increase the risk of mental nerve injury from nerve retraction or even direct injury from surgical instruments. In contrast, our study has shown multi-segmental maxillary procedures did not pose additional risk of NSD. It could be explained by the fact that the segmentalization of maxillary segments was within the dentoalveolar segments and was far from the infra-orbital nerves.

It has been suggested better surgical skill or experience might reduce the risk of NSD after orthognathic surgery [13], [19]. Kobayashi et al. reported a larger proportion of subjects with objective and subjective NSD of lower lip after SSO performed by a group of “surgeons with little experience” when compared to two surgeons with over 100 SSO experiences [13]. However, such correlation was not found in our study when we compared the incidence of NSD after orthognathic surgery performed by surgeons of three levels of experience. We believe the experience of the surgeon may have insignificant effect on NSD and its recovery, provided that surgical procedures are performed properly.

The limitation of this study was the retrospective cross-sectional study design limited the possibility to observe the longitudinal recovery pattern of NSD after orthognathic surgery. There might have bias and confounding factors that might not be avoidable in a retrospective study. The movements of the specific maxillary and mandibular procedures were not reported, which were possible factors that affect the incidence of NSD. We therefore recommend for future research to design a prospective longitudinal study on the recovery pattern of NSD after orthognathic surgery.

## Conclusion

This retrospective cross-sectional study of NSD after orthognathic surgery in a local major orthognathic centre showed the incidence of subjective NSD after maxillary procedures were 16.2%, 13% and 9.8% at post-operative 6 months, 12 months and 24 months, respectively; the incidence of subjective NSD after mandibular procedures were 35.4%, 36.6% and 34.6% at post-operative 6 months, 12 months and 24 months, respectively. Objective neurosensory tests showed general reduced sensitivity in subjects with subjective NSD. Increased age was found to be a significant risk factor of NSD after orthognathic surgery at short term (at 6 months and 12 months) but not at 24 months. SSO has a higher risk of NSD when compared to VSSO. SSO in combination with anterior mandibular surgery has a higher risk of NSD when compared to VSSO in combination with anterior mandibular surgery or anterior mandibular surgery alone. Gender of patients and surgeons’ experience were not found to be risk factors of NSD after orthognathic surgery.
